# Clinical and psychological approaches to the diagnosis of children with autism spectrum disorders

**DOI:** 10.1192/j.eurpsy.2021.586

**Published:** 2021-08-13

**Authors:** A. Koval-Zaytsev, M. Ivanov, N. Simashkova, S. Nikitina

**Affiliations:** Department Of Child Psychiatry, Federal State Budgetary Scientific Institution “Mental Health Research Center”, Moscow, Russian Federation

**Keywords:** autism spectrum disorders, diagnosis, cognitive dysontogenezis

## Abstract

**Introduction:**

The importance of clinical diagnosis of autism spectrum disorders (ASD) in childhood is due to the timely detection of ASD and the appropriate early start of patient care, depending on the form of ASD. The experience of multidisciplinary collaboration between medical psychologists and clinicians in child psychiatric practice allows us to more accurately determine the depth and severity of autistic manifestations, determine the dynamics of child development, and provide personalized effective care.

**Objectives:**

Develop diagnostic, clinical and psychological approaches to the diagnosis of ASD.

**Methods:**

Clinical-psychopathological, clinical-dynamic, clinical-catamnestic, and psychological methods were used. 254 patients aged 4-17 years (average – 7.3 years) with different forms of ASD were examined.

**Results:**

From the clinical and pathopsychological positions, profiles of six main forms of ASD are identified. Each of the selected profiles corresponds to a specific type of cognitive dysontogenezis. A distorted view of cognitive dysontogenezis in Asperger’s syndrome (F84. 5) and childhood autism dysontogenetic (F84.0). Distorted or deficient types of cognitive dysontogenezis in children’s psychosis (F84. 02). Deficient type of cognitive dysontogenezis in Kanner syndrome (F84. 01). Defecating type of cognitive dysontogenezis in atypical autism syndromal (F84. 11), deficient and regressive-defecating types of cognitive dysontogenezis in atypical child psychosis (F84.12).

**Conclusions:**

A three-dimensional model is obtained that allows the most accurate diagnosis of various forms of ASD and the development of personalized routes for patient care and rehabilitation, taking into account the type of cognitive dysontogenezis and based on the zone of the child’s immediate development.

